# Subdural hematoma due to skull base bone metastasis of epidermal growth factor receptor mutated non‐small cell lung cancer

**DOI:** 10.1002/ccr3.9122

**Published:** 2024-07-11

**Authors:** Tomohiko Oka, Naohiro Oda, Shigeru Daido, Ichiro Takata

**Affiliations:** ^1^ Department of Internal Medicine Fukuyama City Hospital Fukuyama Japan; ^2^ Department of Neurological Surgery Fukuyama City Hospital Fukuyama Japan

**Keywords:** lung cancer, Osimertinib, skull base bone metastasis, subdural hematoma

## Abstract

Subdural hematoma due to skull base bone metastasis of lung cancer is rare but are oncological emergency, necessitating prompt identification when a headache develops with the progression of the malignancy.

## 1 CASE PRESENTATION

**FIGURE 1:**
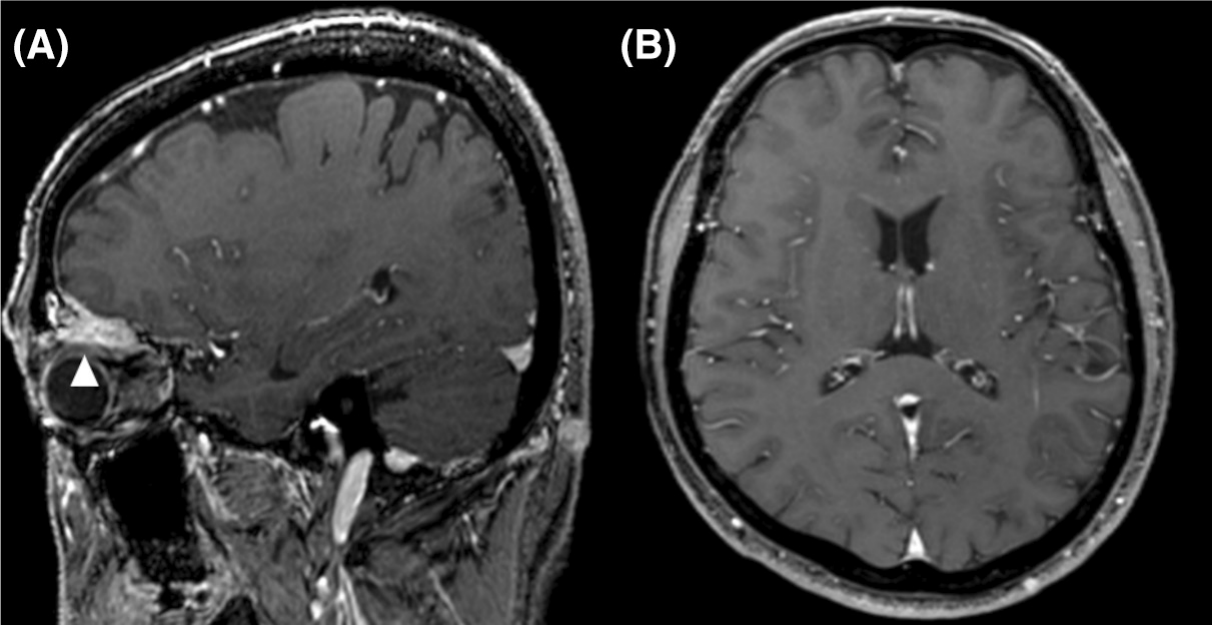
Gadolinium‐enhanced head magnetic resonance imaging indicative of skull base bone metastasis without evidence of hematoma and brain metastasis (A, B).

**FIGURE 2:**
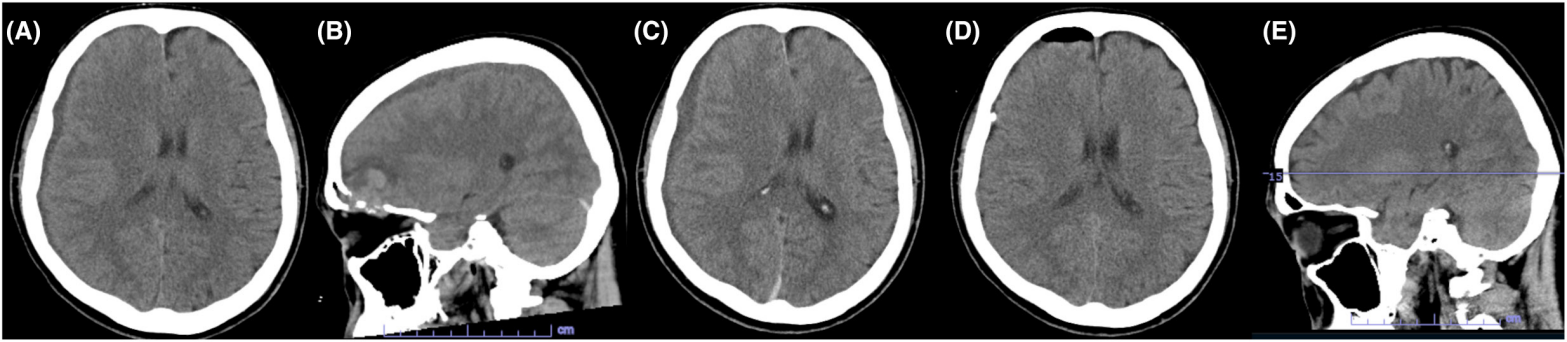
Head computed tomography (CT) scans showed a subdural hematoma communicating the skull base bone metastasis on admission (A, B). Head CT scans showed an increase in hematoma size and an exacerbation of midline shift 9 h after admission (C). Head CT scans showed reduced hematoma and improved midline shift on the second day of hospitalization (D). Head CT showed markedly reduced skull base bone metastasis 2 months after initiating Osimertinib (E).

A man in his 50s presented with chest and back pain lasting for a month, prompting a referral to our hospital because of suspected lung cancer based on computed tomography (CT) findings. He was a former smoker. Contrast‐enhanced CT of the trunk showed a 25 mm nodule in S6 of the right lung and suggestive evidence of multiple metastases to the pancreas, liver, and bones. Gadolinium‐enhanced head magnetic resonance imaging (MRI) showed skull base bone metastasis without evidence of hematoma and brain metastasis (Figure 1A,B). CT‐guided biopsy confirmed that the left rib bone lesion was a lung adenocarcinoma (T1cN1M1c, stage IVB). The acquired tissue samples were genetically analyzed using multigene polymerase chain reaction (PCR) panels (AmoyDx® Pan Lung Cancer PCR Panel). On the subsequent day, he presented to our emergency room with a prolonged severe headache for several hours. He had no history of head trauma and did not take any antiplatelet or anticoagulant agents. Head CT showed a subdural hematoma communicating the skull base bone metastasis (Figure 2A, B), and he was admitted to the high care unit. His level of consciousness worsened 9 h after admission, and repeated head CT showed an increase in hematoma size and an exacerbation of midline shift (Figure 2C). Therefore, he urgently underwent burr hole surgery. A significant improvement in symptoms was observed post‐procedure. Head CT showed reduced hematoma and an improved midline shift on the second day of hospitalization (Figure 2D). Adenocarcinoma cells were detected in aspirated hematoma specimens. On the fifth day of hospitalization, analysis of the multi‐gene PCR panel confirmed the presence of the epidermal growth factor receptor (EGFR) exon 21 L858R mutation. On the same day, treatment with Osimertinib was initiated along with stereotactic radiotherapy (30 Gray/10 fractions) for skull base bone metastasis, which commenced on the eighth day of hospitalization. After an 11‐day hospitalization, the patient was discharged in good condition. A head CT taken 2 months after starting Osimertinib showed a significant reduction in skull base bone metastasis (Figure 2E).

## 2 DISCUSSION

Skull base bone metastases predominantly occur in prostate and breast cancers, with lung cancer representing a relatively rare origin.^1^ The dura varies in thickness according to the site, being thinner in the skull base and sinuses. Skull base bone metastases are thought to invade directly via the thin dura mater, leading to subdural hematomas when intratumoral hemorrhage occurs.^2^ Coagulopathy, especially disseminated intravascular coagulation, can complicate cases of subdural hematoma associated with dural metastases, but this case did not present with coagulopathy.^3^ Adenocarcinoma cells were detected in the subdural hematoma of the patient during the burr hole surgery. However, the disease was successfully managed with Osimertinib, a drug known for its ability to effectively penetrate the central nervous system in patients with EGFR‐mutated non‐small cell lung cancer. Notably, the use of comprehensive multi‐gene PCR panels has significantly expedited the genetic analysis of cancer cells. Despite the patient's critical condition, a shorter examination time and prompt administration of a molecularly targeted drug proved beneficial. Subdural hematoma due to skull base bone metastasis of lung cancer is rare but are oncological emergency, necessitating prompt identification when a headache develops with the progression of the malignancy.

## AUTHOR CONTRIBUTIONS

**Tomohiko Oka:** Conceptualization; writing – original draft. **Naohiro Oda:** Conceptualization; writing – original draft. **Shigeru Daido:** Writing – review and editing. **Ichiro Takata:** Writing – review and editing.

## FUNDING INFORMATION

None.

## CONFLICT OF INTEREST STATEMENT

None declared.

## CONSENT

Written informed consent was obtained from the patient's relative.

## Data Availability

Data sharing is not applicable to this article as no datasets were generated or analyzed during the current study.
